# Glutamate and Lipid Metabolic Perturbation in the Hippocampi of Asymptomatic Borna Disease Virus-Infected Horses

**DOI:** 10.1371/journal.pone.0099752

**Published:** 2014-06-23

**Authors:** Liang Zhang, Yang Lei, Xia Liu, Xiao Wang, Zhao Liu, Dan Li, Peng Zheng, Lujun Zhang, Shigang Chen, Peng Xie

**Affiliations:** 1 Department of Neurology, The First Affiliated Hospital of Chongqing Medical University, Chongqing, China; 2 Institute of Neuroscience, Chongqing Medical University, Chongqing, China; 3 Chongqing Key Laboratory of Neurobiology, Chongqing Medical University, Chongqing, China; 4 Department of Pathology, Faculty of Basic Medicine, Chongqing Medical University, Chongqing, China; 5 Department of Neurology, University-Town Hospital of Chongqing Medical University, Chongqing, China; University of Liverpool, United Kingdom

## Abstract

Borna disease virus (BDV) is a neurotropic, enveloped, non-segmented, negative-stranded RNA virus that infects a wide variety of vertebrate species from birds to humans across a broad global geographic distribution. Animal symptomatology range from asymptomatic infection to behavioral abnormalities to acute meningoencephalitis. Asymptomatic BDV infection has been shown to be more frequent than conventionally estimated. However, the molecular mechanism(s) underyling asymptomatic BDV infection remain largely unknown. Here, based on real-time quantitative PCR and Western blotting, a total of 18 horse hippocampi were divided into BDV-infected (n = 8) and non-infected control (n = 10) groups. A gas chromatography coupled with mass spectrometry (GC-MS) metabolomic approach, in conjunction with multivariate statistical analysis, was used to characterize the hippocampal metabolic changes associated with asymptomatic BDV infection. Multivariate statistical analysis showed a significant discrimination between the BDV-infected and control groups. BDV-infected hippocampi were characterized by lower levels of D-myo-inositol-1-phosphate, glutamate, phosphoethanolamine, heptadecanoic acid, and linoleic acid in combination with a higher level of ammonia. These differential metabolites are primarily involved in glutamate and lipid metabolism. These finding provide an improved understanding of hippocampal changes associated with asymptomatic BDV infection.

## Introduction

Borna disease virus (BDV), the causative agent of Borna disease (BD) that owes its name to the German town of Borna, Saxony where a large number of horses died of a fatal neurologic disease during an 1885 epidemic [1], is a neurotropic, non-cytolytic, enveloped, non-segmented, negative-stranded RNA virus with a genome size of approximately 8.9 kb and replicates and transcribes itself in the nuclei of infected cells [2]. Although classical BD has traditionally occurred in geographically restricted areas in Germany, Austria, Switzerland, and Lichtenstein [3]–[5], signs of BDV infection have been reported in horses and several other mammalian species across a broader range of countries, including Israel, Iran, Japan, Australia, the U.S., and several other European countries [6], suggesting that BDV is more widespread than conventionally believed. Human BDV infection is generally accepted, but BDV's association with psychiatric illness remains controversial [7]–[13].

Clinical manifestations after BDV infection in both naturally- and experimentally-infected animals is species-dependent and virus strain-dependent yet lack uniformity even within a particular host species. BDV infection in horses can result in peracute, acute, or subacute BD with meningoencephalitis. Typical clinical signs of equine BD include simultaneous or consecutive changes in behavior, sensibility, mobility, and autonomic nervous system function [4], [14]. Equine BD histopathology has revealed a severe, non-purulent meningoencephalomyelitis with massive perivascular and parenchymal infiltration [15]–[17] caused by an antiviral CD8 T cell–mediated immune response that results in neurological disease [18], [19]. Since BDV-specific antibodies or RNAs have been found in clinically healthy horses in many geographic areas including China [20]–[24], asymptomatic infection appears to be more frequent than conventionally estimated. However, the underlying mechanism(s) of asymptomatic BDV infection in the horse central nervous system (CNS) have not been adequately characterized.

Metabonomics enables the simultaneous quantitative measurement of numerous low molecular weight molecules within a particular biological sample [25], [26]. Metabolic profiling techniques, such as gas chromatography-mass spectrometry (GC-MS), liquid chromatography-mass spectrometry (LC-MS) [27], and nuclear magnetic resonance (NMR) coupled with multivariate statistical modeling, have been used to analyze the changes in whole metabolic patterns in response to non-physiological challenges such as viral infection [28]–[30]. Our group previously applied a ^1^H-NMR-based metabonomic approach to analyze intracellular metabolic changes in BDV-infected cells [31], which has improved our understanding of the pathogenic mechanisms of BDV.

In this study, a GC-MS-based metabolomic approach was employed to profile and characterize the significantly altered metabolites in the hippocampi of asymptomatic BDV-infected horses relative to non-infected healthy control horses. These finding should provide an improved understanding of the hippocampal changes associated with asymptomatic BDV infection.

## Materials and Methods

### Ethics Statement

This study was performed strictly according to the recommendations of *Guide for the Care and Use of Laboratory Animals* [32]. The sampled animals were restrained humanely by experienced animal care technicians. Euthanasia was performed using a lethal dose of sodium pentobarbital (150 mg/kg) administered intravenously according to the methods described by *AVMA Guidelines on Euthanasia* [33]. All hippocampal samples involved in this study were collected during our previous BDV infection epidemiological study of healthy domestic animals in western China [34]. This study was approved by the Ethics Committee of Chongqing Medical University (Permit number: 20070012).

### Horse Subjects

A total of 18 horses were enrolled in this study and were grazed at the same grassland in Xinjiang, China. The asymptomatic BDV-infected group consisted of eight horses that were both BDV RNA-positive and protein-positive in hippocampal tissues and simultaneously BDV RNA/antibody-positive in blood. The healthy control group consisted of ten horses that were free of BDV RNA and proteins in both hippocampal tissues and blood. In addition, all horses tested negative for common viral infections, including equine herpes virus 1 (EHV-1), rabies virus, and West Nile virus. The healthy control horses were matched by age and sex to the asymptomatic BDV-infected horses. No horse showed neurological abnormalities at the time of sampling.

### Hippocampal Tissue Preparation

Hippocampal samples were obtained from horses post-euthanasia. Briefly, the brain was removed by handsaw with a three-cut technique to remove the calvaria. The skull was pried off using a screwdriver. The horse's nose was elevated, and the olfactory bulbs and cranial nerves were severed while tipping the brain out of the braincase. All removed brain tissue specimens were dissected on dry ice. Isolated hippocampal samples were immediately immersed in liquid nitrogen until they were transferred to a freezer (−80°C).

### Reverse Transcription Real Time Quantitative PCR (RT-qPCR) Detection of BDV p24 and p40 RNA

Total RNA was extracted from hippocampal samples with Trizol reagents (Life Technologies, Gaithersburg, USA) according to the manufacturer's protocols. RNA were simultaneously isolated from hippocampal tissues of persistently BDV strain V-infected rats and healthy rats that served as positive and negative controls, respectively. RNA samples were dissolved in 25 µl of Dnase/RNase-free H_2_O and stored at −80°C until later use. Isolated RNA was reverse transcribed into cDNA using the Reverse Transcriptase System (Promega, Madison, USA). Real-time quantitative PCR (RT-qPCR) was performed as previously described [35] for detection. Briefly, PCR amplification was performed in 25 µl volumes per well, and the PCR procedural steps were followed as previously described. All reactions were run in a Corbett Research Rotor-Gene 6000 thermo cycler (Corbett Research, Mortlake, Australia). Cq values greater than 35 were deemed negative. Finally, the positive RT-qPCR products were cloned to the pGEM-T Easy vector system (Promega, Madison, USA), and the cloned products were sequenced at a commercial facility (Invitrogen Corp., Shanghai, China) by the Applied Biosystems Prism dye-terminator dideoxy system. The sequencing result was used as a query sequence in a BLAST search at GenBank to confirm the specificity of RT-qPCR.

### Western Blot Detection of BDV p24 and p40 Proteins

Total proteins were extracted from homogenated hippocampal tissues using a total protein extraction kit (KeyGEN Biotech, Nanjing, China). Hippocampal protein samples were diluted with 0.1 M phosphate-buffered solution (PBS) and 5× loading buffer to a protein concentration of 1 µg/µl and then heated at 99°C for 5 min. Samples (20 µg/lane) were run on a 16% tricine gel as previously described [36], and the proteins were transferred to polyvinylidene fluoride (PVDF) membranes (Millipore). Then, the membranes were blocked in a 5% (w/v) skimmed milk solution for 3 h at room temperature and incubated overnight at 4°C with mouse monoclonal anti-p24 or anti-p40 (diluted 1∶300, Genscript, Nanjing, China). After three 30-min washings in 100 mM Tris–HCl buffer (pH 7.5) with 150 mM NaCl and 0.05% Tween 20 (TTBS buffer), the membranes were incubated at 37°C for 60 min with goat anti-mouse immunoglobulin G (IgG) (1∶10000; KPL, Gaithersburg, MD,USA) and washed three times for 30 min with TTBS buffer. The membranes were developed with the enhanced chemiluminescence (ECL) reagent Luminata Crescendo Western HRP Substrate (Millipore Corporation, Billerica, USA), and the chemiluminescence signal was then visualized with X-ray film. Hippocampal tissues from persistently-infected BDV strain V rats and healthy rats processed in the same manner were used as positive and negative controls, respectively.

### Tissue Sample Processing for GC/MS Analysis

The homogenized hippocampal samples were submerged in a solution consisting of chloroform–methanol–water (2∶5∶2, v/v/v). The whole mixture was sonicated for 60 min. Subsequently, the samples were centrifuged at 18000 g for 15 min, then 50 µl of ^13^C_6_-leucine was added as an internal standard to 100 µl of supernatant. The collected supernatant was evaporated under a stream of nitrogen gas to complete dryness. A total of 30 µl methoxamine hydrochloride pyridine (20 mg/ml) was added to each dried residue sample, vortex-mixed for 30 s, and incubated at 37°C for 90 min with persistent shaking. Then, we added 30 µl of bis-(trimethylsilyl)–trifluoroacetamide (BSTFA) with 1% trimethylchlorosilane (TMCS) to each sample, and the mixture was left to incubate for 1 h at 70°C.

### GC/MS Analysis

The procedure of GC/MS analysis was performed as previously described [37]. Briefly, the analysis was performed on an Agilent 7890A/5975C GC/MS system (Agilent, Santa Clara, CA, USA) equipped with a HP-5MS fused silica capillary column (30 m×0.25 mm×0.25 µm; Agilent J&W Scientific, Folsom, CA, USA). The ultra-pure helium was used as carrier gas at a constant flow rate of 1 ml/min through the column. The injector temperature was set at 280°C. The column temperature was initially maintained at 80°C for 2 min and then increased from 80 to 320°C at 10°C/min with a hold time of 6 min. The column effluent was introduced into the ion source of an Agilent 5973 mass selective detector (Agilent Technologies). The MS quadrupole temperature was set at 150°C and the ion source temperature at 230°C. The electron energy was 70 eV, and mass data were collected in full scan mode (m/z 50–600).

### Statistical Analysis

All raw files from GC/MS were converted into NetCDF (network Common Data Form format) via TagFinder [38]. This processing enabled deconvolution, alignment, and data reduction to produce a list of mass and retention time pairs with corresponding intensities for all detected peaks from each data file in the data set. Normalization was performed in Microsoft Excel prior to multivariate analysis. The resulting three-dimensional matrix, involving peak index (RT-m/z pair), sample names (observations), and normalized peak area percent, were introduced into the SIMCA-P 11.0 software package (Umetrics AB, Umeå, Sweden) in order to utilize principal component analysis (PCA) to display natural separation between the experimental and control groups by visual inspection of score plots.

Then, pair-wise orthogonal projections to latent structures discriminant analyses (OPLS-DA) [39] was performed to identify metabolites contributing to the differences between the two groups. Subsequently, a two-tailed Student's *t*-test was performed for validation at the univariate analysis level while the discriminating metabolites were obtained using a statistically significant threshold of variable influence on projection (VIP) values. A *p*<0.05 and VIP>1.0 were considered statistically significant. Finally, the selected metabolites were identified by comparing the mass fragments with those present in the commercial mass spectral databases for qualitative analysis.

## Results

### Identification of BDV Infection in Hippocampal Tissue

**Figure 1:**
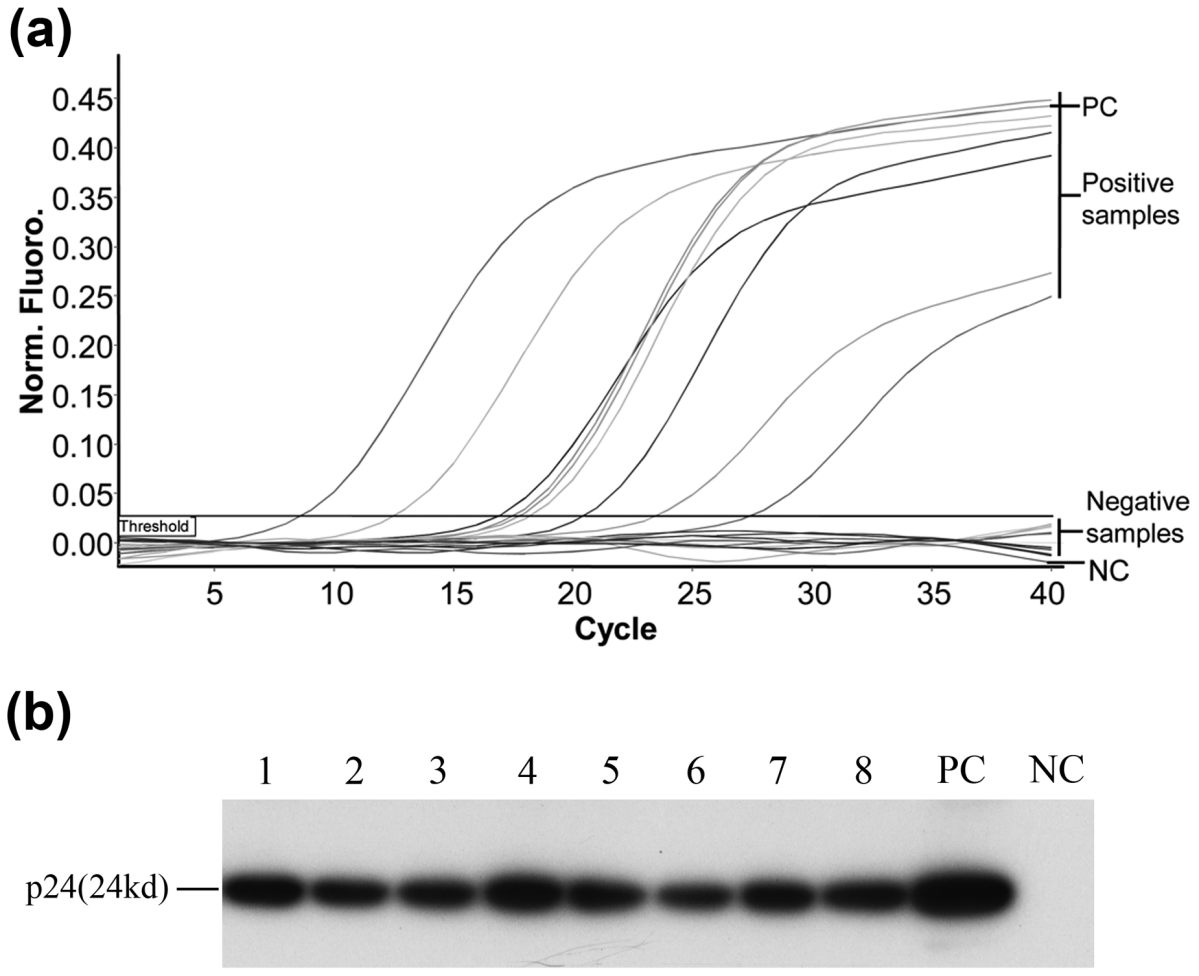
BDV Detection in Horse Hippocampal Tissues by RT-qPCR and Western Blotting. Amplification plots of RT-qPCR for BDV p24 RNA from all samples are showed (A). The cycle number on the horizontal axis is plotted against the normalized fluorescence on the vertical axis. Signals were regarded as positive if and only if the fluorescence intensity exceeded 10 times the standard deviation of the baseline fluorescence (threshold). Cq values greater than 40 were regarded as negative. BDV p24 proteins in all hippocampal samples were detected by Western blotting (B). Lanes 1–8, p24 protein positive tissue. PC: positive control sample, hippocampal tissue from persistently BDV-infected rat. NC: negative control sample, hippocampal tissue from healthy rat.

For RT-qPCR assays, negative control samples in each assay were consistently negative, and Cq values of positive control samples were less than 25 cycles. These findings indicate that the RT-qPCR system functioned normally. All sequencing results of amplicon revealed a greater than 95% identity with GenBank's BDV p24 or p40 fragment. As a result, RT-qPCR and Western blot assays indicated that the eight BDV-infected horses' hippocampal tissues were both BDV p24 and p40 RNA-positive and protein-positive. The ten horses in the healthy control group all tested negative for BDV p24 and p40 RNA and protein (Fig. 1)

### Metabolomic Analysis

**Figure 2:**
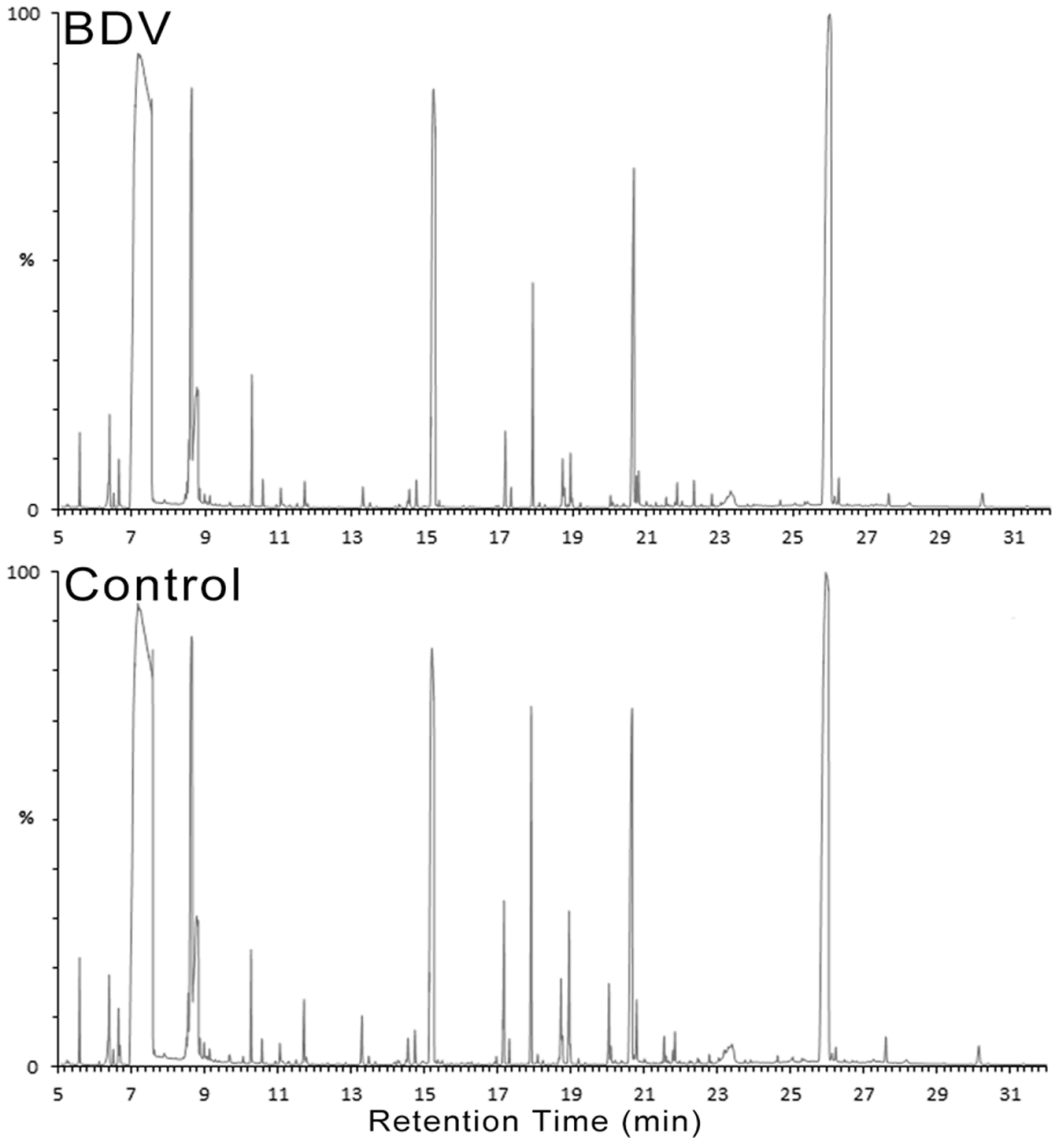
Representative GC-MS Total Ion Current Chromatograms from BDV-Infected and Control Horses.

**Figure 3:**
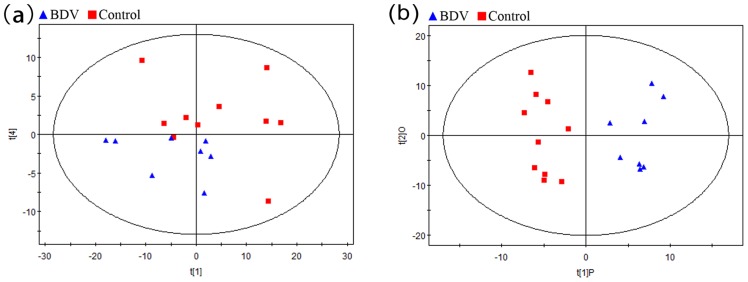
2D-PCA Scores Map (PC1 versus PC4) (A) and 2D Cross-Validated OPLS-DA Score Map (B) of GC-MS Data. Each dot denotes an individual sample (BDV-infected, n = 8; control, n = 10). The ellipse represents the Hotelling's T2 with 95% confidence in score plots.

Representative GC/MS total ion current (TIC) chromatograms of hippocampal samples from the BDV and control group are displayed in Fig. 2. Initially, we took advantage of 2D-PCA scores plot (principal components 1 versus principal components 4) in order to reflect the metabolic differences associated with BDV infection. Although the clusters of the BDV and control groups partially overlapped, distinct clustering of metabolic profiles was still obtained (Fig. 3a). The OPLS-DA model, a supervised projection method, verified a better class separation (Fig. 3b), and *R^2^Y* and *Q^2^* demonstrated high robustness (*R^2^Y* = 0.918, *Q^2^* = 0.732).

### Significantly Differentiated Metabolites

In the current study, a GC/MS-based metabolomic approach was utilized to independently distinguish the hippocampal metabolic signatures of asymptomatic BDV-infected horses and healthy controls. Based on the VIP threshold of the OPLS-DA model, a total of six significant variables were ultimately obtained (Table 1). Subsequently, a two-tailed Student's *t*-test with a threshold *p*-value of 0.05 was performed to determine significant differences on a metabolite-by-metabolite basis. Compared to the control group, the BDV-infected group was characterized by lower levels of D-myo-inositol-1-phosphate, glutamate, phosphoethanolamine, heptadecanoic acid, and linoleic acid in combination with higher level of ammonia, revealing that BDV infection primarily alters glutamate and lipid metabolism in the hippocampi of the asymptomatic BDV-infected horses.

**Table 1:** Differential Metabolites between BDV-Infected and Control Groups.

Compound	VIP value*	*P*-value†	Fold change§
D-myo-inositol-1-phosphate	1.33	0.02261	−0.51
Glutamate	1.33	0.04145	−1.75
Phosphoethanolamine	1.60	0.00996	−2.31
Heptadecanoic acid	1.40	0.02854	−0.31
Linoleic acid	1.59	0.01119	−0.45
Ammonia	1.97	0.00073	0.96

*Variable importance in the projection (VIP) value obtained from the OPLS model with a cutoff of 1.0.

† *P*-value calculated using a two-tailed Student's t-test (significance at *p*<0.05).

§ A positive fold change indicates a relatively higher concentration in the BDV-infected group as compared to controls, while a negative value indicates a relatively lower concentration in the BDV-infected group as compared to controls.

## Discussion

### D-myo-inositol-1-phosphate

D-myo-inositol-1-phosphate is an intermediate of the inositol phosphate metabolism pathway. Under the action of phosphohydrolase, D-myo-inositol-1-phosphate is converted into myo-inositol. Alzheimer's disease researchers have reported that increased myo-inositol accompanies inflammation expressed as reactive microglial activation and reactive astrocytosis, which is a well-known pathological response typically observed in neurodegenerative diseases and also a hallmark of BDV-infected neonatal rodent and equine brains [40]–[43], [17]. Thus, the decreased levels of D-myo-inositol-1-phosphate observed in the BDV-infected group here may be associated with increased myo-inositol consumption during glial activation.

### Glutamate

Glutamate, a primary excitatory neurotransmitter in the mammalian CNS, was found to be significantly decreased in the BDV-infected group relative to the control group. BDV's affinity for glutamatergic neurons, including the granule neurons of the dentate gyrus, has been previously reported [44], [45]. The axons of CA3 pyramidal neurons carry viral antigens to the stratum oriens and stratum radiatum of the CA1 region, but the BDV does not appear to spread from the axon terminals to the CA1 neurons. As these neurons are heavily loaded with BDV proteins, severe morphological and functional damage of the glutamatergic system may ensure, resulting in decrease of intracerebral glutamate synthesis. Based on the glutamate deficiency hypothesis, this phenomenon may explain why persistent human BDV infection may contribute to the development of depressive symptoms [46], [47].

### Lipid Metabolites

Phosphoethanolamine – a precursor of phosphatidylethanolamine, a major component of the lipid membrane [48] – was significantly decreased in the BDV-infected group relative to the control group. We also observed significant decreases in two fatty acids, heptadecanoic acid and linoleic acid, in the BDV-infected group relative to the control group. Similarly, our previous GC-MS metabolomic study of BDV Hu-H1 infection in the rat hippocampus revealed significant perturbations in several lipid metabolites including cholesterol, myristic acid, and 1-monopalmitoylglycerol [49].

## Conclusions

This study reveals that changes in glutamate and lipid metabolism occur in the hippocampus of asymptomatic BDV-infected horses. These finding provide an improved understanding of hippocampal changes occurring in asymptomatic BDV infection. Further study on the mechanism(s) underlying transformation from asymptomatic BDV infection to symptomatic BDV infection are necessary.

## References

[1] Ludwig H, Bode L (2000) Borna disease virus: new aspects on infection, disease, diagnosis and epidemiology. Rev Sci Tech 19: 259–288. 11189720 10.20506/rst.19.1.1217

[2] De la Torre JC, Bode L, Carbone K, Dietzschold B, Ikuta K, et al.. (2000) Family Bornaviridae. In: Van Regenmortel MHV, Fauquet CM, Bishop DHL, editors. Virus Taxonomy: Classification and Nomenclature of Viruses: Seventh Report of the International Committee on Taxonomy of Viruses. London, England: Academic Press. pp. 531–538.

[3] Ludwig H, Thein P (1977) Demonstration of specific antibodies in the central nervous system of horses naturally infected with Borna disease virus. Med Microbiol Immunol 163: 215–226. 604728 10.1007/BF02125505

[4] Ludwig H, Kraft W, Kao M, Gosztonyi G, Dahme E, et al (1985) Borna virus infection (Borna disease) in naturally and experimentally infected animals: its significance for research and practice. Tierarztl Prax 13: 421–453. 3834641

[5] Rott R, Becht H (1995) Natural and experimental Borna disease in animals. Curr Top Microbiol Immunol 190: 17–30. 7789148 10.1007/978-3-642-78618-1_2

[6] Kinnunen PM, Palva A, Vaheri A, Vapalahti O (2013) Epidemiology and host spectrum of Borna disease virus infections. J Gen Virol 94: 247–262. 23223618 10.1099/vir.0.046961-0

[7] Rott R, Herzog S, Fleischer B, Winokur A, Amsterdam J, et al (1985) Detection of serum antibodies to Borna disease virus in patients with psychiatric disorders. Science 228: 755–756. 3922055 10.1126/science.3922055

[8] Bode L, Zimmermann W, Ferszt R, Steinbach F, Ludwig H (1995) Borna disease virus genome transcribed and expressed in psychiatric patients. Nat Med 1: 232–236. 7585039 10.1038/nm0395-232

[9] Bode L, Durrwald R, Rantam FA, Ferszt R, Ludwig H (1996) First isolates of infectious human Borna disease virus from patients with mood disorders. Mol Psychiatry 1: 200–212. 9118344

[10] Sauder C, Muller A, Cubitt B, Mayer J, Steinmetz J, et al (1996) Detection of Borna disease virus (BDV) antibodies and BDV RNA in psychiatric patients: evidence for high sequence conservation of human blood-derived BDV RNA. J Virol 70: 7713–7724. 8892892 10.1128/jvi.70.11.7713-7724.1996 PMC190841

[11] Bode L, Reckwald P, Severus WE, Stoyloff R, Ferszt R, et al (2001) Borna disease virus-specific circulating immune complexes, antigenemia, and free antibodies–the key marker triplet determining infection and prevailing in severe mood disorders. Mol Psychiatry 6: 481–491. 11443538 10.1038/sj.mp.4000909

[12] Bode L, Ludwig H (2003) Borna disease virus infection, a human mental-health risk. Clin Microbiol Rev 16: 534–545. 12857781 10.1128/CMR.16.3.534-545.2003 PMC164222

[13] Thakur R, Sarma S, Sharma B (2009) Role of Borna disease virus in neuropsychiatric illnesses: are we inching closer? Indian J Med Microbiol 27: 191–201. 19584498 10.4103/0255-0857.53200

[14] Richt JA, Rott R (2001) Borna disease virus: a mystery as an emerging zoonotic pathogen. Vet J 161: 24–40. 11145828 10.1053/tvjl.2000.0533

[15] Gosztonyi G, Ludwig H (1984) Borna disease of horses. An immunohistological and virological study of naturally infected animals. Acta Neuropathol 64: 213–221. 6437125 10.1007/BF00688111

[16] Joest E (1911) Untersuchungen über die pathologische Histologie, Pathogenese und postmortale Diagnose der seuchenhaften Gehirn-Rückenmarksentzündung (Bornaschen Krankheit) des Pferdes. Ein Beitrag zur vergleichenden Pathologie des Zentralnervensystems. Journal of Neurology 42: 293–324.

[17] Priestnall SL, Schoniger S, Ivens PA, Eickmann M, Brachthauser L, et al (2011) Borna disease virus infection of a horse in Great Britain. Vet Rec 168: 380b. 10.1136/vr.c6405 21498268

[18] Bilzer T, Stitz L (1993) Brain cell lesions in Borna disease are mediated by T cells. Arch Virol Suppl 7: 153–158. 10.1007/978-3-7091-9300-6_12 8219800

[19] Planz O, Bilzer T, Stitz L (1995) Immunopathogenic role of T-cell subsets in Borna disease virus-induced progressive encephalitis. J Virol 69: 896–903. 7815558 10.1128/jvi.69.2.896-903.1995 PMC188657

[20] Bahmani MK, Nowrouzian I, Nakaya T, Nakamura Y, Hagiwara K, et al (1996) Varied prevalence of Borna disease virus infection in Arabic, thoroughbred and their cross-bred horses in Iran. Virus Res 45: 1–13. 8896237 10.1016/0168-1702(96)01355-x

[21] Hagiwara K, Asakawa M, Liao L, Jiang W, Yan S, et al (2001) Seroprevalence of Borna disease virus in domestic animals in Xinjiang, China. Vet Microbiol 80: 383–389. 11348775 10.1016/s0378-1135(01)00324-8

[22] Herzog S, Frese K, Richt J, Rott R (1994) Ein Beitrag zur Epizootiologie der Bornaschen Krankheit beim Pferd. WIENER TIERARZTLICHE MONATSSCHRIFT 81: 374–374.

[23] Kinnunen PM, Billich C, Ek-Kommonen C, Henttonen H, Kallio RK, et al (2007) Serological evidence for Borna disease virus infection in humans, wild rodents and other vertebrates in Finland. J Clin Virol 38: 64–69. 17129759 10.1016/j.jcv.2006.10.003

[24] Nakamura Y, Kishi M, Nakaya T, Asahi S, Tanaka H, et al (1995) Demonstration of Borna disease virus RNA in peripheral blood mononuclear cells from healthy horses in Japan. Vaccine 13: 1076–1079. 7491814 10.1016/0264-410x(95)00050-b

[25] Beckonert O, Keun HC, Ebbels TM, Bundy J, Holmes E, et al (2007) Metabolic profiling, metabolomic and metabonomic procedures for NMR spectroscopy of urine, plasma, serum and tissue extracts. Nat Protoc 2: 2692–2703. 18007604 10.1038/nprot.2007.376

[26] Nicholson JK, Lindon JC, Holmes E (1999) ‘Metabonomics’: understanding the metabolic responses of living systems to pathophysiological stimuli via multivariate statistical analysis of biological NMR spectroscopic data. Xenobiotica 29: 1181–1189. 10598751 10.1080/004982599238047

[27] Zhou B, Xiao JF, Tuli L, Ressom HW (2012) LC-MS-based metabolomics. Mol Biosyst 8: 470–481. 22041788 10.1039/c1mb05350g PMC3699692

[28] Munger J, Bajad SU, Coller HA, Shenk T, Rabinowitz JD (2006) Dynamics of the cellular metabolome during human cytomegalovirus infection. PLoS Pathog 2: e132. 17173481 10.1371/journal.ppat.0020132 PMC1698944

[29] Sripadi P, Shrestha B, Easley RL, Carpio L, Kehn-Hall K, et al (2010) Direct detection of diverse metabolic changes in virally transformed and tax-expressing cells by mass spectrometry. PLOS ONE 5: e12590. 20830293 10.1371/journal.pone.0012590 PMC2935367

[30] Sitole LJ, Williams AA, Meyer D (2013) Metabonomic analysis of HIV-infected biofluids. Mol Biosyst 9: 18–28. 23114495 10.1039/c2mb25318f

[31] Huang R, Gao H, Zhang L, Jia J, Liu X, et al (2012) Borna disease virus infection perturbs energy metabolites and amino acids in cultured human oligodendroglia cells. PLOS ONE 7: e44665. 22970281 10.1371/journal.pone.0044665 PMC3436876

[32] Resources ILA (1996) Guide for the Care and Use of Laboratory Animals. Washington, DC: National Academy Press. 125 p.

[33] Association APoEAVM (2001) 2000 Report of the AVMA Panel on Euthanasia. J Am Vet Med Assoc 218: 669–696. 11280396 10.2460/javma.2001.218.669

[34] Zhang L, Wang X, Zhan Q, Wang Z, Xu M, et al (2014) Evidence for natural Borna disease virus infection in healthy domestic animals in three areas of western China. Arch Virol doi: 10.1007/s00705-013-1971-5 10.1007/s00705-013-1971-5 24573218

[35] Schindler AR, Vogtlin A, Hilbe M, Puorger M, Zlinszky K, et al (2007) Reverse transcription real-time PCR assays for detection and quantification of Borna disease virus in diseased hosts. Mol Cell Probes 21: 47–55. 17014984 10.1016/j.mcp.2006.08.001 PMC7127217

[36] Schägger H (2006) Tricine–SDS-PAGE. Nat Protoc 1: 16–22. 17406207 10.1038/nprot.2006.4

[37] Shao W, Fan S, Lei Y, Yao G, Chen J, et al (2013) Metabolomic identification of molecular changes associated with stress resilience in the chronic mild stress rat model of depression. Metabolomics 9: 433–443.

[38] Luedemann A, Strassburg K, Erban A, Kopka J (2008) TagFinder for the quantitative analysis of gas chromatography-mass spectrometry (GC-MS)-based metabolite profiling experiments. Bioinformatics 24: 732–737. 18204057 10.1093/bioinformatics/btn023

[39] Cloarec O, Dumas ME, Trygg J, Craig A, Barton RH, et al (2005) Evaluation of the orthogonal projection on latent structure model limitations caused by chemical shift variability and improved visualization of biomarker changes in 1H NMR spectroscopic metabonomic studies. Anal Chem 77: 517–526. 15649048 10.1021/ac048803i

[40] Eisenman LM, Brothers R, Tran MH, Kean RB, Dickson GM, et al (1999) Neonatal Borna disease virus infection in the rat causes a loss of Purkinje cells in the cerebellum. J Neurovirol 5: 181–189. 10321982 10.3109/13550289909022000

[41] Hornig M, Weissenbock H, Horscroft N, Lipkin WI (1999) An infection-based model of neurodevelopmental damage. Proc Natl Acad Sci U S A 96: 12102–12107. 10518583 10.1073/pnas.96.21.12102 PMC18419

[42] Kreutzberg GW (1996) Microglia: a sensor for pathological events in the CNS. Trends Neurosci 19: 312–318. 8843599 10.1016/0166-2236(96)10049-7

[43] Siger M, Schuff N, Zhu X, Miller BL, Weiner MW (2009) Regional myo-inositol concentration in mild cognitive impairment Using 1H magnetic resonance spectroscopic imaging. Alzheimer Dis Assoc Disord 23: 57–62. 18725861 10.1097/WAD.0b013e3181875434 PMC3039549

[44] Gosztonyi G, Ludwig H (1995) Borna disease–neuropathology and pathogenesis. Curr Top Microbiol Immunol 190: 39–73. 7789150

[45] Gosztonyi G, Ludwig H (2001) Interactions of viral proteins with neurotransmitter receptors may protect or destroy neurons. Curr Top Microbiol Immunol 253: 121–144. 11417131 10.1007/978-3-662-10356-2_6

[46] Gosztonyi G (2008) Natural and experimental Borna disease virus infections–neuropathology and pathogenetic considerations. APMIS Suppl: 53–57. 10.1111/j.1600-0463.2008.000m8.x 18771099

[47] Belmaker RH, Agam G (2008) Major depressive disorder. N Engl J Med 358: 55–68. 18172175 10.1056/NEJMra073096

[48] Kano-Sueoka T, Cohen DM, Yamaizumi Z, Nishimura S, Mori M, et al (1979) Phosphoethanolamine as a growth factor of a mammary carcinoma cell line of rat. Proc Natl Acad Sci U S A 76: 5741–5744. 293677 10.1073/pnas.76.11.5741 PMC411726

[49] Lei Y, Li D, Deng J, Shao W, Fan S, et al (2014) Metabolomic profiling of three brain regions from a postnatal infected Borna disease virus Hu-H1 rat model. Metabolomics 10: 484–495.

